# Historical efforts to develop ^99m^Tc-based amyloid plaque targeting radiotracers

**DOI:** 10.3389/fnume.2022.963698

**Published:** 2022-08-16

**Authors:** Ghazaleh Takalloobanafshi, Aditi Kukreja, Justin W. Hicks

**Affiliations:** ^1^Department of Chemistry, Western University, London, ON, Canada; ^2^Cyclotron and Radiochemistry Facility, Lawson Health Research Institute, London, ON, Canada; ^3^Department of Medical Biophysics, Western University, London, ON, Canada; ^4^Saint Joseph's Health Care London, London, ON, Canada

**Keywords:** technetium, Alzheimer's disease, radiotracer development, rural and remote access, single photon emission computed tomography (SPECT)

## Abstract

Imaging biomarkers have changed the way we study Alzheimer's disease and related dementias, develop new therapeutics to treat the disease, and stratify patient populations in clinical trials. With respect to protein aggregates comprised of amyloid-β plaques and tau neurofibrillary tangles, Positron Emission Tomography (PET) has become the gold standard imaging modality for quantitative visualization. Due to high infrastructural costs, the availability of PET remains limited to large urban areas within high income nations. This limits access to leading edge medical imaging, and potentially access to new treatments, by millions of rural and remote residents in those regions as well as billions of people in middle- and low-income countries. Single Photon Emission Computed Tomography (SPECT) is a more widely available imaging alternative with lower infrastructural costs and decades of familiarity amongst nuclear medicine professionals. Recent technological advances have closed the gap in spatial resolution and quantitation between SPECT and PET. If effective SPECT radiotracers were available to visualize amyloid-β plaques, geographic barriers to imaging could be circumvented. In this review, we will discuss past efforts to develop SPECT radiotracers targeting amyloid-β plaques which incorporate the most used radionuclide in nuclear medicine: technetium-99m (^99m^Tc; *t*_1/2_ = 6.01 h; γ = 140 keV). While reviewing the various chemical scaffolds and chelates employed, the focus will be upon the impact to the pharmacological properties of putative ^99m^Tc-based amyloid-targeting radiotracers.

## Introduction

Alzheimer's disease (AD) and related dementias have a significant socioeconomic impact on patients, their families, and communities. If the economic burden of AD were a country, it would be the 18th largest in the world, estimated to surpass two trillion US dollars this decade and affects nearly 55 million people worldwide (1, 2). This number is expected to double every 20 years with a new case diagnosed approximately every 3 s (3). Much of this growth will be in low- and middle-income counties where access to diagnoses and therapies may be hindered. Even in high income countries, barriers remain to rural and remote communities to accessing the best care. This is particularly dire as diagnosis and therapies transition from symptomatic (sitting with physician) to measuring biological and genetic biomarkers (fluid and imaging).

As knowledge of the underlying biological processes driving AD grows, researchers have been able to identify fluid and imaging biomarkers to detect changes in the brain before symptoms begin to manifest with a research framework from the National Institute on Aging and Alzheimer's Association (NIA-AA) recently redefining AD to include biomarkers (4). Although not intended for general clinical practice, the research framework does provide a strong scaffold for such guidance. Significant focus is placed upon the protein aggregates of amyloid beta (Aβ) as extracellular plaques, tau as neurofibrillary tangles, and neuronal injury such as metabolic or perfusion changes (so-called A/T/N classification) (5). Recent promising results with plasma biomarkers, particularly pTau217, may make large impacts on clinical diagnosis and disease staging (6, 7), however, they are limited to systemic and not direct measurements within the brain. Imaging has a powerful role to play in visualizing and quantifying these biomarkers non-invasively through application of positron emission tomography (PET) and single photon emission computed tomography (SPECT).

Recent advances in nuclear medicine imaging of AD and kindred dementias have taken center stage within the NIA-AA research framework. Several PET radiopharmaceuticals labeled with ^11^C and ^18^F (*t*_1/2_ = 20.4 and 109.8 min) have been approved by regulatory bodies and licensed for clinical use (8). One such example is [^18^F]florbetapir (9), which was recently used to demonstrate reduced amyloid burden following treatment with aducanumab (Aduhelm; the first approved anti-amyloid drug) (10). These promising results could change clinical management of AD patients; however, in order to receive this therapy, patient need to first have a PET scan to assess amyloid burden. High infrastructure and operating costs have limited availability of PET to large population centers. This in turn has hindered the broad implementation of PET beyond a research setting. Consequently, rural, remote, and developing populations without available PET biomarker imaging have difficulty accessing improved AD therapies.

**Figure 1:**
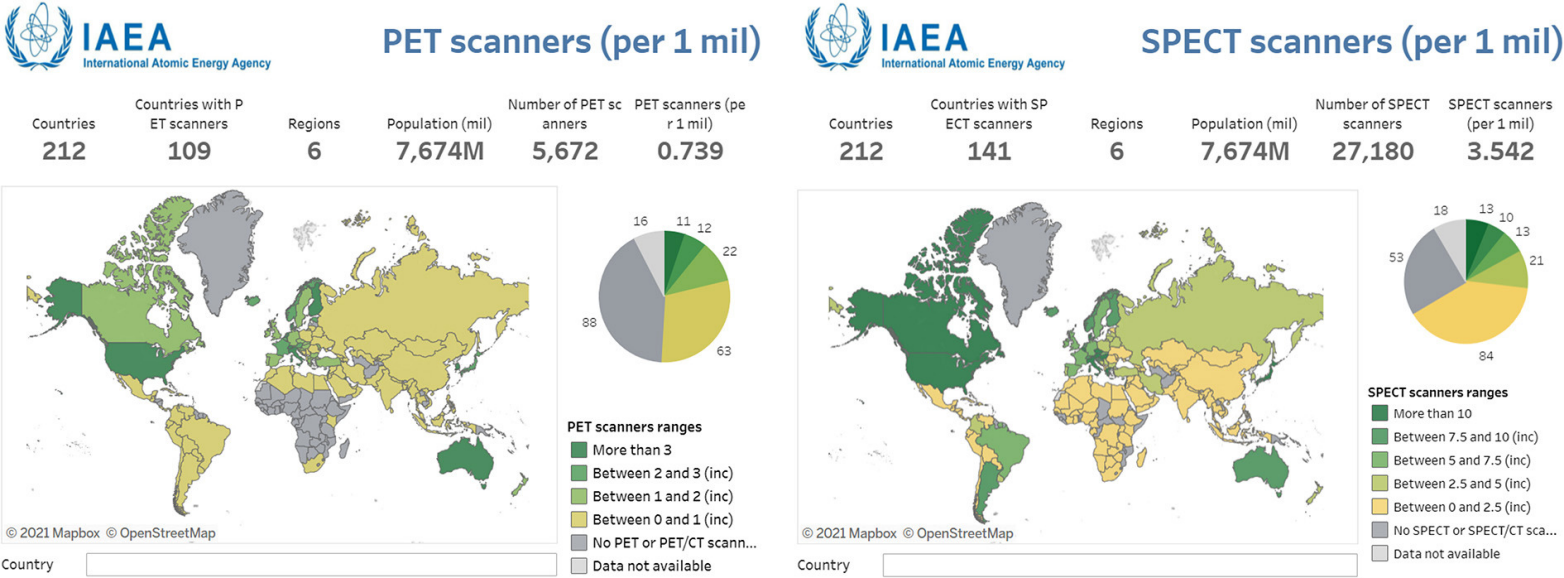
Numbers of PET and SPECT scanners per million people displayed across a global map. In every instance, SPECT is far more accessible or the sole option for nuclear medicine imaging. Reproduced from the IAEA IMAGINE database (14). Note the change in scale between two images.

The other nuclear medicine imaging technology, SPECT, has been widely available since the early 1990s. As of 2015 in Canada, SPECT scanners outnumber PET scanners by at least 40:1 (11, 12). In a vast, sparsely populated country, access to either of these highly sensitive modalities could improve patient management, reduce burden on patients and caregivers, and improve therapy outcomes (13). More broadly speaking, the International Atomic Energy Agency established the IMAGINE database in 2012 to illustrate the availability of medical imaging and nuclear medicine worldwide (14). A similar trend to Canada is seen worldwide, with Figure 1 showing higher accessibility to SPECT compared to PET. Much of this increased access can be attributed to lower infrastructure costs (15). While a PET scanner costs circa $2 million, SPECT systems can be purchased for around $600,000. As discussed in more detail below, neither of these systems can operate without radiotracers. SPECT also has an advantage here with typically longer half-life (i.e., longer use per production and greater distribution range) and lower radionuclide production costs compared to cyclotron production required for PET. More information on access and availability of radiopharmaceuticals can be found in a recent review of the topic (16).

In neurology application of nuclear medicine, PET has dominated due to the plethora of small molecule radiotracers labeled with ^11^C and ^18^F which target proteins of interest in psychiatric and neurological disorders (17). Furthermore, it was assumed the spatial resolution and relative ease for quantification with PET would ensure its dominance over SPECT (18). In recent reviews focused on advancements to SPECT technology, brain SPECT received barely a mention (19, 20). Another perspective paper focused on the future direction of molecular imaging failed to even mention (21) or barely mentioned (22) SPECT as a modality. Many technological advances were made over the past 10 years, particularly cadmium zinc telleride detectors (23), novel collimator designs (24), improved reconstruction (25), and flexible ring gantries (26). These have drastically closed the gap in spatial resolution (2.5 mm on clinical or submillimeter on preclinical scanners) (23), quantitation (±5% of true radionuclide concentration) (27), and sensitivity [up to 850 count/s/MBq for CZT-based SPECT (28)]. These improvements mean SPECT is now matching or surpassing PET with respect to scanner performance. Even with older SPECT cameras, the modality has been shown to be more cost effective in some cases compared to PET (e.g., the SPARC trial) (29). It is time to reconsider assumptions regarding SPECT to improve access to molecular neuroimaging. To fully exploit these technological improvements, advancements are required in radio-pharmacy and -chemistry.

The two primary radionuclides used for neurological SPECT are iodine-123 (^123^I; *t*_1/2_ = 13.2 h) and technetium-99m (^99m^Tc; *t*_1/2_ = 6.01 h). Both have been used clinically to study neurodegenerative diseases. Hexamethyl propylene oxime (HMPAO) coordinated to ^99m^Tc has been used in brain perfusion imaging in AD for decades (30). These SPECT perfusion studies found changes in brain regions associated with neurodegeneration similar to [^18^F]FDG-PET hypometabolism. Importantly, these areas of hypoperfusion were not impacted by blood glucose levels. Given co-morbidities between diabetes and AD, perfusion SPECT may be a more reliable biomarker in these patients (31). As for targeted radiotracers, tropane-based probes for dopamine transporters have had the most clinical success. Although not used for imaging AD, [^123^I]FP-CIT (Datscan) is used frequently to image dopaminergic tone in Parkinson's disease (32). As ^123^I is not readily available in low- and middle-income countries (33), an alternative tropane for dopamine transporters, [^99m^Tc]Trodat, represents the only clinically approved, targeted ^99m^Tc-based radiotracer (34–36). The success of [^99m^Tc]Trodat holds promise that a ^99m^Tc-based amyloid radiotracer is achievable.

The focus of this primer review will be on developments over the past two decades toward producing an effective ^99m^Tc-based radiotracer targeting Aβ aggregates. This topic has been previously reviewed as part of broader, metal-based probe discussions (37–40), or focused on Tc alone (41). Our goal is to update the field and provide the most comprehensive collection of *in vitro* and *ex vivo* data to date. Discussion will focus upon *in vitro* binding affinity and selectivity, blood-brain barrier (BBB) penetration, and pharmacokinetic data in wild-type and transgenic animal models. Although no radiotracer has emerged for human use, it remains worthwhile to pursue a simple, inexpensive, and highly accessible imaging method for the non-invasive quantification of proteinopathies in neurodegeneration. Such a tool could have major repercussions for the understanding of the pathogenesis of AD and also for its diagnosis and treatment (42, 43).

## Radiotracers of Aβ plaques based on ^99m^Tc

Given the wider availability of SPECT technology, it is past time for expanding the library of readily available probes for neuroimaging. There are several examples of ^123^I being successfully employed for neuro-SPECT (44) but logistical and chemical challenges remain (45, 46). As the most used radionuclide in nuclear medicine, radiopharmacies are familiar with the chelation chemistry and regularly handle ^99m^Tc (47). The wide availability from a ^99^Mo/^99m^Tc generator and ideal imaging properties for SPECT make ^99m^Tc-based targeted probes desirable.

Getting a metal-based radiotracer through the blood-brain barrier is a non-trivial task. The chelates used to attach the metal to the targeting vector (i.e., antibody, peptide, small molecule, etc.) often have a large impact on *in vivo* properties. Choosing a targeting vector-chelate pair with appropriate lipophilicity, overall size, and polarity mitigates these impacts. The addition of the chelate should also have a minimal impact upon the affinity and selectivity of the radiotracer to maintain the biological properties of the targeting vector. Any putative Tc-based radiotracers must demonstrate an ability to differentiate Aβ binding in AD patients from age-matched controls. Off-target binding, tissue clearance, metabolism, and pharmacokinetics matching the physical and biological half-lives of the radiotracer also play a crucial role in obtaining an effective SPECT imaging agent. Additionally, a practical production method is required for either point of care formulation or large-scale production and distribution. These ideal properties have been reviewed extensively elsewhere and readers are referred to these for more information (44, 46, 48).

### Earliest attempts

**Figure 2:**
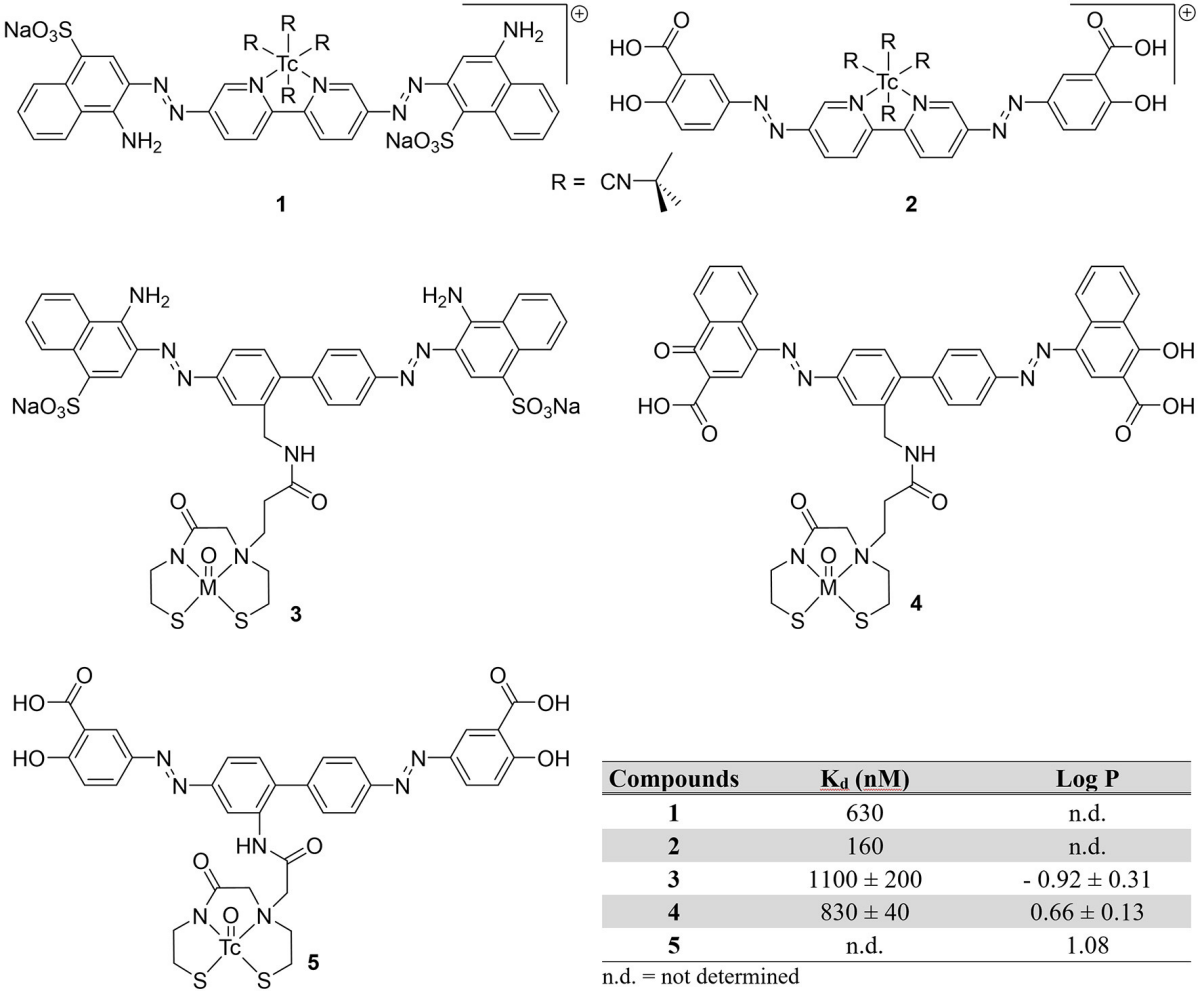
Chemical structures of earliest attempts to develop a ^99m^Tc-based probe for amyloid plaques.

Prior to 2000, only three attempts to image Aβ plaques with ^99m^Tc were conducted (Figure 2). It was believed that conjugating a ^99m^Tc chelate to a common histological dye would make a moderate radiotracer to image amyloid plaques. By substituting pyridinyl rings for phenyl groups in the central portion of the dyes Congo Red in compound **1** and Chrysamine G in compound **2**, rhenium or technetium could be chelated (49). Previous studies showed this would have minimal impact on binding affinity, since binding is strongly driven by flat, extended pi systems fitting between Aβ sheets (50). Han et al. labeled these modified dyes with ^99^Tc (*t*_1/2_ = 2.1 × 10^5^ years; β^−^) in 28 and 29% yields, respectively. Their binding affinities for Aβ_1−40_ were determined to be 630 and 160 nM for **1** and **2**. Since both chelates were charged, it was assumed there was limited utility for brain imaging without assistance opening the BBB.

Congo Red and a derivative dye were later modified with a monoamine monoamide bis thiol (MAMA') chelates (**3** and **4**) (42). These chelates were conjugated with Re for *in vitro* testing and **3** was also labeled with ground state ^99^Tc in 88% yield. The binding affinities for the isosteric Re congeners were comparable to Congo Red (K_d_ = 1,500 ± 0.005 nM) in the authors' assay. When administered to brain tissue slices, amyloid in both blood vessels and plaques were visualized with both compounds, albeit with **Re-4** in 2.5 higher concentration. Additionally, the charged nature of **3**, large size (MW > 1,000 Da) and hydrophilicity make BBB penetration unlikely.

In a final twentieth century report, Chrysamine G was conjugated to ^99m^Tc through a MAMA chelator by the Leuven group (51). With the help of microwave heating, ^**99m**^**Tc-5** was obtained in 75% yield. They were able to obtain promising *in vitro* properties (log P = 1.08, >4 h stability) along with some selectivity for Aβ in autoradiography experiments with brain tissue slices from AD patients. When compared to ^125^I-labeled human serum albumin, there was slightly higher brain:blood ratios (indicative of BBB permeability), however, biodistribution studies in healthy mice (male NMRI mice, no age given) were not promising. There was very low initial brain uptake (0.3 ± 0.1 %ID/g at 2 min post-injection or p.i.) with no activity observed at 60 min p.i. The rapid clearance from blood and increasing activity in the intestinal tract could be an indication of *in vivo* metabolism.

There was little chance for success penetrating the BBB using these large and/or charged dye molecules. When it comes to brain radiotracers, BBB permeability is considered a crucial factor. In addition to affinity and selectivity for Aβ aggregates, candidate SPECT radiotracers must also optimize lipohilicity, polar surface area, size, and proton donor/acceptor pairs to pass through the BBB. Several types of molecular transport mechanisms have been identified at the BBB (52). It's commonly accepted that non-polar, lipid-soluble molecules can passively diffuse through the cell membranes of endothelial cells across the BBB and enter the brain. Second, receptor-mediated transport mechanism, allowing polar nutrients (i.e., carbohydrates, metal ions, and amino acids) and molecular carriers (such as transferrin) to enter the brain. Conversely, efflux transporters work to remove exogenous molecules from the brain. Testing for efflux transporter affinity can rule out this mechanism for poor brain uptake.

Following the early success of PET radiotracers based on smaller, neutral dyes such as thioflavin S, benzofurans, hydrazines, etc., ^99m^Tc-based probes began following a similar trend. The following sections are divided based upon chelating class—conjugated or integrated. Each of these is broken down further by targeting vector into various groups: (1) benzo-furan and (2) benzo-thiazoles (3) stilbenes, (4) chalcones, and (5) miscellaneous. Several additional compounds using bivalent ligand structures are also included.

## Conjugated probes

There are two main chelator classifications which group the putative SPECT radiotracers. The first is based on the classical tethered ^99m^Tc-chelate with the targeting vector and metal complex separated by a linker. It is thought that by separating the bulky metal-ligand complex, the affinity to target will be unaffected by the added volume (53). As consequence of adding carbon or ethoxy chains, the mass and lipophilicity are altered. Although most examples below maintained adequate affinity for Aβ aggregates *in vitro*, the added mass and often higher lipophilicity values may have negatively contributed to poor biophysical attributes.

### Benzothiazoles

#### For cerebral Aβ detection

**Figure 3:**
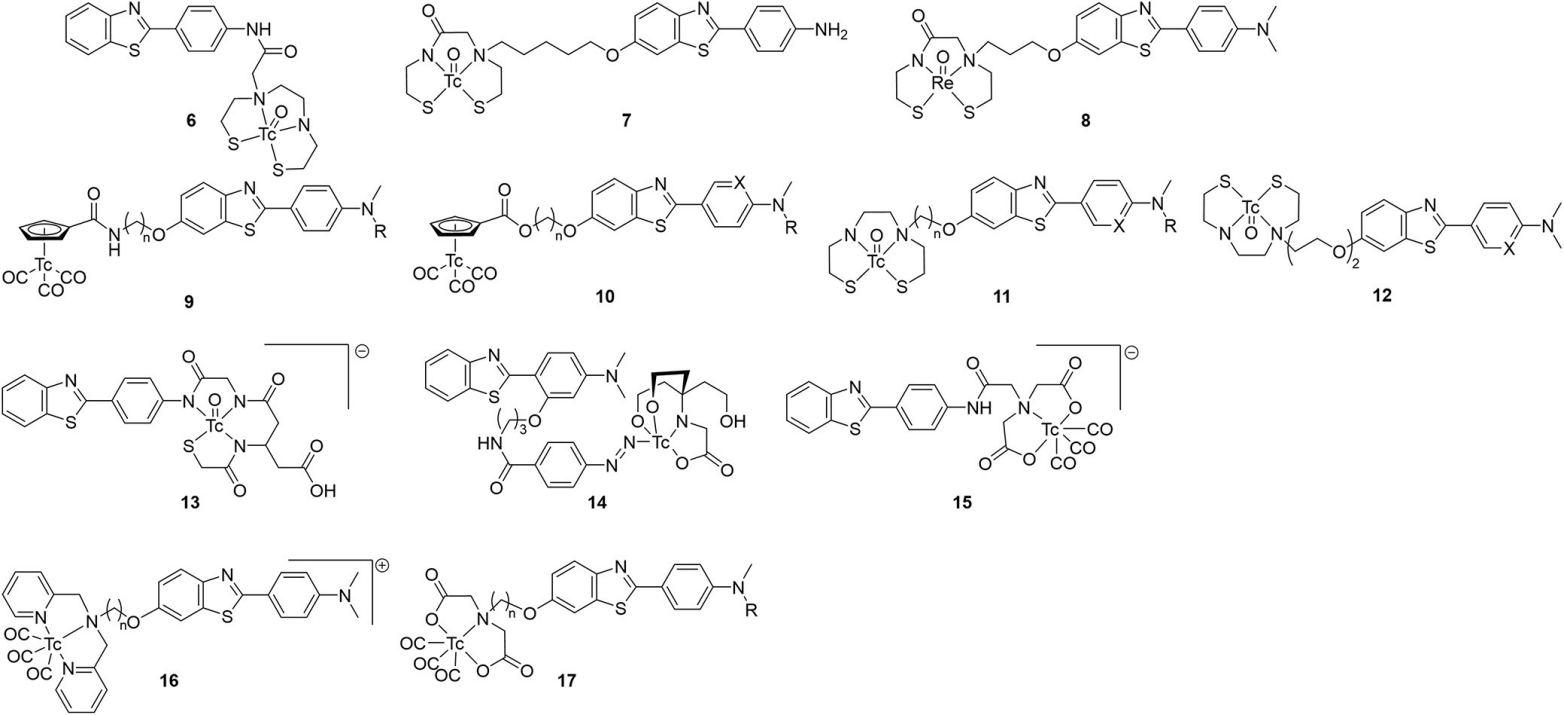
Chemical scaffolds of ^99m^Tc-labeled benzothiazole aniline derivatives for imaging cerebral Aβ plaques.

With the surge of PET radiotracer development culminating in the success of [^11^C]Pittsburgh Compound B (PiB), there was seemly a hiatus from ^99m^Tc-based radiotracer development from 2000 to 2006. In 2007, the Leuven group reported the first ^99m^Tc benzothiazole candidate (Figure 3; Table 1) (54). They chelated ^99m^Tc *via* a bis-amino-bis-thiol (BAT) conjugated to the aniline of a thioflavin-T derivative in a mean yield of 86%. By adding carrier ^99^Tc, the group was able to obtain mass spectroscopic data confirming the presence of **6** (m/z = 559.2), confirming the complexation of [Tc(V)O]^3+^. The affinity for Aβ plaques was assessed qualitatively using postmortem brain slices with and without the presence of thioflavin-T (1 μM). Any blocking of the binding was indicative of similar binding sites between **6** and thioflavin-T. Along with the low mass (<600 Da) and moderate lipophilicity, **6** was investigated in normal mice (stain, sex, and age not given) for brain uptake. The early uptake was low (0.9 and 0.11% ID in the cerebrum and cerebellum, respectively) and the compound was deemed unsuitable for detecting amyloid deposition in living brains. Moreover, by analyzing the mouse plasma it was found that by 2 min p.i., 85% of the injected activity was metabolized to a polar metabolite, leaving little intact **6** to cross the BBB.

**Table 1:** *In vitro* and *ex vivo* properties of ^99m^Tc-labeled benzothiazole aniline derivatives for imaging cerebral Aβ plaques.

							**% Injected dose/gram (%ID/g)**		
**Compound**		***n***	**R**	**X**	***K*_i_ (nM)**	**Log P**	**2 min**	**60 min**	**Ratio^a^**
**6**					n.d.	1.92 ± 0.012	0.09	0.03	3.00
**7**					n.d.	2.90 ± 0.13	1.34 ± 0.16	0.65 ± 0.10	2.01
**8**					10.0	3.26		n.d.	
**9**	**a**	3	H		143 ± 26	3.51 ± 0.014	0.50 ± 0.10	0.18 ± 0.07	2.78
	**b**	5	H		76 ± 37	3.17 ± 0.04	0.36 ±0.07	0.19 ± 0.02	1.89
	**c**	3	Me		64 ± 14	3.55 ±0.10	0.26 ± 0.04	0.11 ± 0.02	2.36
	**d**	5	Me		24 ± 8	3.26 ± 0.06	0.37 ± 0.08	0.14 ± 0.03	2.64
**10**	**a**	3	H	CH	19 ± 3	2.69 ± 0.08	1.06 ± 0.16	0.39 ± 0.06	2.72
	**b**	3	Me	CH	13 ± 5	2.99 ± 0.12	0.70 ± 0.14	0.17 ± 0.03	4.12
	**c**	3	H	N	204 ± 34	2.81 ± 0.02	0.69 ± 0.08	0.31 ± 0.12	2.23
	**d**	3	Me	N	134 ± 25	2.81 ± 0.02	0.54 ± 0.10	0.10 ± 0.03	5.40
**11**	**a**	4	Me	CH	16 ± 6	n.d.	0.69 ± 0.16	0.46 ± 0.12	1.50
	**b**	5	Me	CH	34 ± 18	n.d.	0.46 ± 0.09	0.40 ± 0.09	1.15
	**c**	6	Me	CH	8 ± 1	n.d.	0.59 ± 0.12	0.43 ± 0.15	1.37
	**d**	4	H	CH	9 ± 2	n.d.	2.11 ± 0.11	0.62 ± 0.08	3.40
	**e**	5	H	CH	29 ± 14	n.d.	0.92 ± 0.09	0.63 ± 0.15	1.46
	**f**	5	Me	N	18 ± 13	n.d.	0.47 ± 0.07	0.19 ± 0.03	2.47
	**g**	6	Me	N	24 ± 3	n.d.	0.60 ± 0.05	0.29 ± 0.05	2.07
**12**	**a**			CH	31 ± 6	2.88 ± 0.04	1.57 ± 0.15	0.54 ± 0.21	2.91
	**b**			N	103 ± 31	2.48 ± 0.06	0.92 ± 0.24	0.15 ± 0.03	6.13
**13**					n.d.	−0.42 ± 0.02	0.28 ± 0.08	0.20 ± 0.10	1.40
**14**					n.d.	0.81 ± 0.01	0.38 ± 0.07	0.017 ± 0.05	2.24
**15**					n.d.	1.1 ± 0.02	0.07 ± 0.02	0.03 ± 0.01	2.33
**16**	**a**	3			162 ± 43	n.d.		n.d.	
	**b**	5			37 ± 17	2.80 ± 0.25	0.18 ± 0.02	0.08 ± 0.02	2.25
**17**	**a**	4	H		45 ± 12	n.d.	0.80 ± 0.17	0.03 ± 0.01	26.67
	**b**	6	H		62 ± 8	n.d.	0.61 ± 0.08	0.18 ± 0.01	3.39
	**c**	4	Me		51 ± 13	n.d.	0.88 ± 0.14	0.14 ± 0.02	6.29
	**d**	6	Me		42 ± 11	n.d.	1.21 ± 0.22	0.06 ± 0.01	20.17

n.d., not determined.

a Clearance ratio of 60 min/2 min.

The following year, a group at Beijing Normal tethered a MAMA ligand to the hydroxy on the benzothiazole and evaluated **7** as a potential β-amyloid SPECT probe (55). Following on earlier computational work, this site was selected to minimize impact on binding affinity if the linker is sufficiently long (56). The Re congener was shown to bind to brain tissue from transgenic mice (C57 APP, 12 months, no sex given) and a human AD patient (male, 57 yo). Once complexed with ^99m^Tc, the biodistribution of **7** in normal mice (IRC, no age or sex given) was investigated. Moderate initial uptake (1.34 ± 0.16 %ID/g) with slow wash-out was observed (0.65 %ID/g at 60 min p.i.). As no Aβ plaques were expected in these normal mice, a roughly 50% reduction in brain uptake points to a high level of non-specific binding. Furthermore, the high amount of **7** remaining in blood even at 2 h p.i. (4.71 ± 1.24 %ID/g) is problematic for imaging.

In 2009, the originators of PiB prepared six Re complexes of 2-phenylbenzothiazoles which were never radiolabeled (57). These compounds were conjugated through pendant group (**8**) or integrated into the molecular structure to reduce the overall size (**43**–**45**; see below). Biological evaluation of the conjugated compound **8** showed strong binding affinity (K_i_ = 10 nM) and reasonable lipophilicity (Log P = 3.26) for permeating the BBB. Although **8** had the highest affinity in this group of putative SPECT radiotracers, the three-carbon tether added to the molecular weight (>600 g/mol compared to < 500 for integrated examples below) and the lipophilicity (integrated were all < 2.60 compared to 3.26). Analogous compounds **11** and **12** were radiolabeled by other groups, as discussed below.

Hoping to minimize the impact the chelator had upon brain uptake, Jia et al. elected to use a piano-stool cyclopentadienyl Tc(I) tricarbonyl core for compounds **9** and **10** (58, 59). The CpM(CO)_3_ ligand has several advantages including low molecular weight, compact size, minimal steric impact, and moderate lipophilicity. Importantly, these half-sandwich complexes are highly stable. In their first study, the linker length and number of methyl groups of **9** were varied, with slighty lower affinity compared to a common assay standard [^125^I]IMPY (K_i_ = 12.5 ± 2.8 nM in this report) (58). Tissue from 12-month-old, female, C57BL6 APPswe/PSEN1 transgenic mice was use for fluorescent staining and autoradiographic experiments which showed co-localization to thioflavin-S (or the absence with age-matched controls). In normal mice (ddY, 5-week-old, male) there was unsatisfactory initial brain uptake (0.26–0.50 %ID/g) and poor wash-out (2 min/60 min %ID/g ranged from 1.9 to 2.8). This poor *in vivo* performance was attributed to high overall molecular weight and the amide bond of the linker which is available to form hydrogen bond. The follow-up study with **10** changed the link to an ester and added a heteroatom to the aniline moiety (59). These changes had a positive impact on binding affinity but did not significantly improve the brain penetration or clearance. Although this group argues the conjugated approach, tethering the chelate through a linker, is needed to achieve high affinity, later work will demonstrate the benefits of integrating the CpM(CO)_3_ group into the targeting vector (vide infra).

Sticking with the conjugated approach, the Beijing group next reported further derivatization of the 2-anilino-benzothiazole core (**11**; Figure 3) (60). Switching back to BAT for chelating ^99m^Tc, a subset of these compounds had as good or better binding affinity compared to [^125^I]IMPY for Aβ_1−42_ aggregates. Those with the lowest K_i_ were radiolabeled and evaluated *ex vivo* in normal mice (ICR, male, no age given). Fluorescent staining with the Re congeners distributed with a similar pattern to thioflavin-S with AD patient and transgenic mouse (C57BL6 APPswe/PSEN1, male, 12-months-old) brain slices. This gave some evidence for specificity. Compound **11d** had the highest initial brain uptake reported to date with reasonable clearance. The study's authors propose the lower lipophilicity (as assigned by HPLC retention times) of **11d** led to less plasma protein binding. They concede there are other factors which could have led to higher brain uptake which were not explored in the present study. Nonetheless, the encouraging *ex vivo* results led to the first *in vivo* use of a ^99m^Tc radiotracer candidate in non-human primates. Semi-quantitative analysis of the SPECT/CT images revealed low initial brain uptake of **11d** (1.23 and 0.78 %ID in a healthy male and female rhesus monkey, respectively) with slow clearance (0.88 and 0.64 %ID at 40 min p.i., respectively). As one of the most comprehensive studies reviewed, this exemplary paper should act as a guide for anyone entering this field.

As part of a follow up study, Zhang et al. altered the linker moiety of **12** to an oligoethyleneoxy to further modify the lipophilicity (61). This did not have the desired effect on binding affinity as the values increased compared to the alkyl chain. There was also a diminished performance in *ex vivo* biodistribution experiments (ICR mice, male, no age given) with lower uptake compared to the promising **11d** (Table 1). Given the better properties of a single methyl on the aniline, it was surprising that these derivatives were not prepared.

#### For cerebrovascular Aβ detection

Three compounds, **13**–**15** (Figure 3; Table 1), were reported as candidates for detecting systemic amyloidosis which may find use in imaging cerebral amyloid angiopathy (CAA) (62). All three bore a 2-phenylbenzothiazole core with different bifunctional chelators used: a mercaptoacetyltriglycine (MAG3; **8**), a hydrazinonicotinic acid (HYNIC)/tricine (**9**), and an imino-diacetic acid (**10**). The first and third were chosen for their negative charge which was hypothesized to limit the hepatobiliary uptake in favor of renal secretion. When used with a tricine co-ligand, Tc-HYNIC complexes are also polar and shown to have similar favorable excretion characteristic, as seen with ^99m^Tc-octreotide derivatives (63). The three candidates were radiolabeled in high yields (80, 70, and 64%, respectively) and evaluated for biodistribution in normal mice (male NMRI, no age given). Given their hydrophilic nature, these compounds unsurprisingly showed low brain uptake (Table 1). Unlike the previous studies, this paper did not report any affinity information or brain tissue binding, likely due to the intended peripheral application. Even so, it was concluded that **13**–**15** had less than ideal pharmacokinetic characteristics, postulated to be from rapid *in vivo* metabolism.

In 2014 the Beijing Normal team produced positively charged dipyridylamine-[^99m^Tc]Tc(CO)_3_ compounds **16** (64). Both compounds, with 3 or 5 carbon chain linkers, had moderate *in vitro* affinity to Aβ_1−42_ but Aβ_1−40_, the predominate peptide in CAA, was not tested. Given a four-fold higher affinity, only **16b** was radiolabeled. It was hypothesized that the larger chelate required more distance from the 2-phenylthiazole moiety for binding. *Ex vivo* biodistribution in normal mice (ICR, male, 5 weeks) indeed showed little brain uptake at 2 or 60 min. As such, **16b** may be a potential SPECT radiotracer for CAA.

Concurrent with their above efforts, the group from Beijing Normal also explored imino-diacetic-^99m^Tc chelates **17** for use with cerebrovascular amyloid (65). Possessing a negative charge will prohibit BBB entry, thus, visualizing only the vascular Aβ deposits is another way to differentiate between AD and cerebral amyloid angiopathy. This polar chelate had a negative impact on binding affinity which was minimally improved by moving the group further from the targeting vector. Although the goal was not to show good brain penetration, surprisingly these compounds did exhibit some of the best clearance in mouse brains (ICR, 5-week-old, male). Compounds **17a** and **17d** both had comparable initial uptake at 2 min p.i. vs. the other compounds in Table 1 but the washout was much greater. This may be due to the wider margin of error (circa 20%) at the early timepoints as the amount of radioactivity present in blood was high (22 and 32 %ID/g for **15a** and **15d)**. The blood volume present for each sample at the time of counting may play a significant role in the variation.

### Benzoxazole and benzofuran

**Figure 4:**
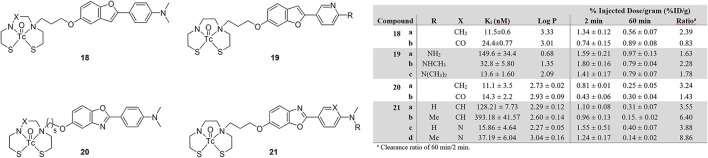
Chemical scaffolds of ^99m^Tc-labeled benzofuran and benzoxazole derivatives for imaging Aβ plaques.

Slightly deviating from the thioflavin dyes, Ono et al. prepared two benzofuran-based ^99m^Tc probes with BAT and MAMA (**18a** and **18b**; Figure 4) chelates (66). In an [^125^I]IMPY based inhibition assay, both compounds bound to Aβ_1−42_ aggregates with the K_i_ of **Re-18a** determined to be lower than **Re-18b**. This affinity was also seen in autoradiographic experiments with **18a** using sections of Tg2576 mouse brains. Observed binding was comparable to staining adjacent slices with thioflavin-S. When investigated for biodistribution in wild-type mice (ddY, 5 weeks, no sex given), **18b** had very low and slow uptake with no washout, making it unviable for SPECT imaging. Although **18a** had moderate initial uptake, the washout was still only about 60% after an hour.

In follow up work on these benzofurans, Cheng et al. changed the aniline to a pyridine and varied the number of methyl groups of compound **19** (67). This structure was based upon previous success by the Kyoto group with [^18^F]FPYBF-2 as a selective PET radiotracer for Aβ plaques (68). The group chose the use a BAT chelator and forgo MAMA as the former is known to have good BBB penetration (69). From Figure 4, comparing the derivatives of **18** and **20** reinforces this decision. Three Re derivatives of **19** were assessed for binding to Aβ_1−42_ using an [^125^I]IMPY assay. Only **19c** retained similar affinity (K_i_ = 13.6 ± 1.60 nM) to IMPY and PiB (K_i_ = 10.5 ± 1.05 and 9.00 ± 1.31 nM, respectively). These results were consistent with increasing affinity correlated to increasing methylation seen in previous studies, however, they were somewhat surprisingly higher than the fluorinated versions (68). The radiolabeled analogs underwent autoradiographic analysis using tissue from Tg2576 and healthy age-matched mice (male, 27-months-old). By comparing to co-stained (thioflavin-S) slices, selectivity for Aβ plaques was shown. This higher affinity and selectivity did not translate to high brain uptake in normal mice (ddY, male, 5-weeks-old). For comparison, [^18^F]florbetapir has >7 %ID/g in the mouse brain (70). More disheartening is the poor washout after 60 min, with 45% remaining in the best case of **19b**.

Structurally similar benzoxazoles **20ab** were reported by Wang et al. albeit with longer linker between the BAT and MAMA chelates (71). The Re congeners were again used to obtain the Aβ_1−42_ binding affinity information. Much like **18a**, the BAT derivative **20a** had slightly stronger binding compared to **20b** (Figure 4) and both were comparable to the value obtain for IMPY (K_i_ = 10.1 ± 1.9 nM) in the same assay. Selectivity for amyloid plaques was determined using brain tissue slices from 12-month-old C57BL6, APPswe/PSEN1 transgenic mice (no sex given). Both compounds clearly stained Aβ plaques with a similar staining pattern to thioflavin-S. To assess the initial brain uptake and clearance, normal 5-week-old mice (no strain or sex given) were used in biodistribution experiments. Neither derivative of **20** had good initial uptake but the prospects of **20b** were further harmed by practically no wash-out. These attempts are therefore not suitable for further evaluation as amyloid targeting SPECT radiotracers.

Follow up studies by the same group led to **21** to improve the biological properties of the benzoxazoles (72). Fluorescent staining with the four Re complexes and thioflavin-S showed consistent co-localization in brain sections from AD patient tissue and transgenic mice (C57BL6 APPswe/PSEN1, 11-months-old, male). Autoradiography with the mouse tissue and the four ^99m^Tc-labeled **21** derivatives also revealed similar distribution of Aβ plaques to thioflavin-S stained sections. For *in vitro* binding affinity, only one compound, **21c**, was comparable to [^125^I]IMPY (K_i_ = 15.9 vs. 11.5 nM). By comparing four **21** derivatives, the presence of the pyridine group improved the binding affinity. Despite this affinity and reasonable lipophilicity, no derivative had sufficient brain uptake, however, the washout of the pyridinyl derivatives **21c** and **21d** was greatly improved with almost 90% of the latter cleared from the mouse brain. Interestingly, two methyl groups on the amine lowered the affinity whereas higher order substitution led to an increase with **19**.

### Miscellaneous fused rings—Flavones, aurones, napthalenes, and phenylquinoxalines

**Figure 5:**
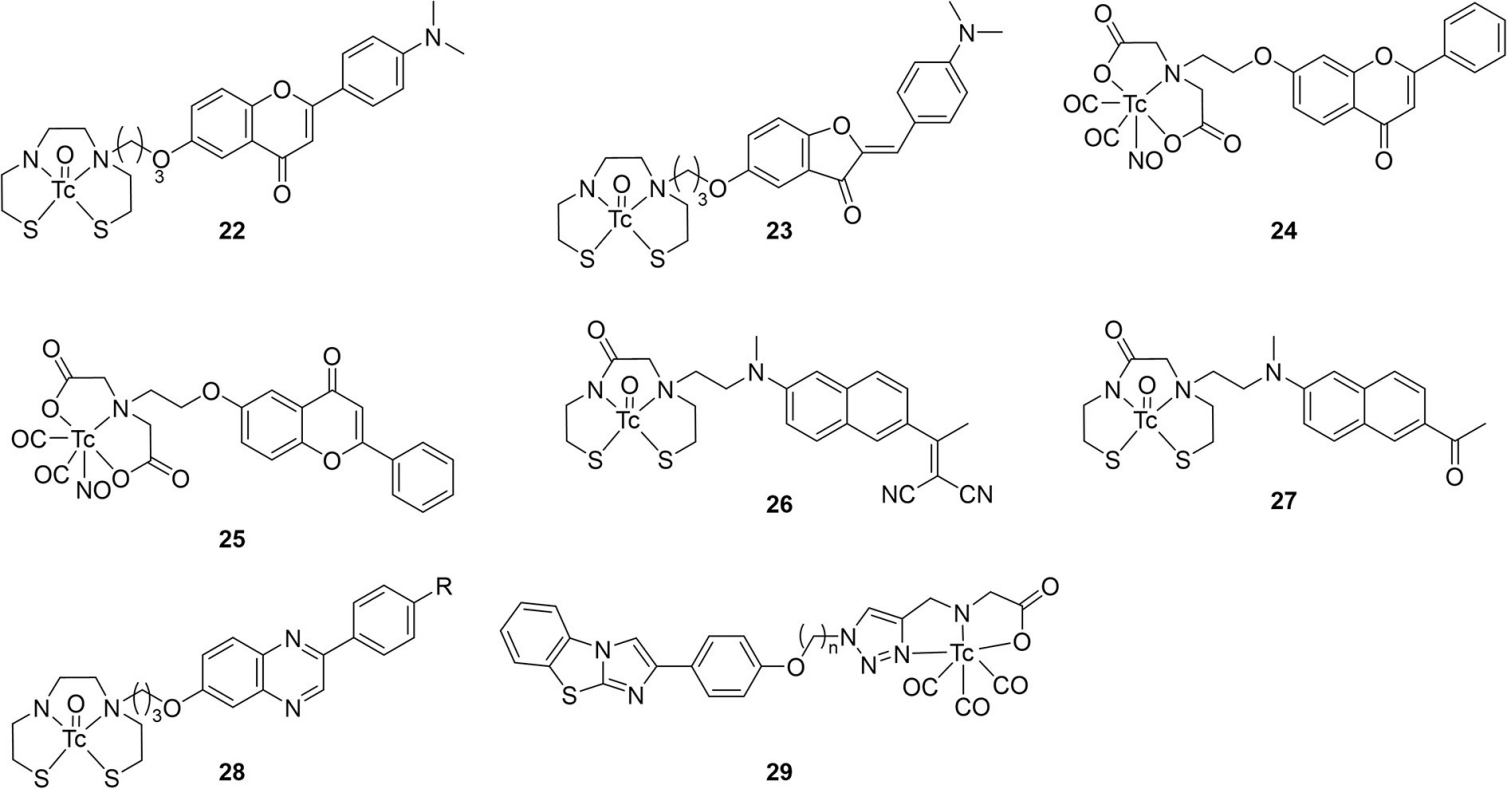
Chemical scaffolds of miscellaneous ^99m^Tc-labeled fused (hetero)cycles.

Beyond the fused heterocycles above, ^99m^Tc-labeled small molecules based upon other planar, extended pi systems were also explored over the past two decades (Figure 5; Table 2). Two early examples, **22** and **23**, come from the Kyoto group (73). These ^99m^Tc complexes based upon aurone and flavone employed BAT chelators to hold the radiometal. Both compounds bound to Aβ_1−42_ aggregates in a dose dependent manner with reported low non-specific binding (<2%). No K_i_ values were determined but the % bound at various concentrations were reported. To confirm **22** and **23** had affinity for Aβ plaques, histochemical staining with their Re congeners and thioflavin-S were carried out with mouse brain sections (Tg2567, 30-months-old, female). The separate staining experiments labeled the Aβ plaques in a similar pattern. The two radiolabeled compounds were then evaluated in *ex vivo* biodistribution experiments in normal mice (ddY, 5-weeks-old, no sex given). Both **22** and **23** were minimally brain penetrate but the little that did enter were mostly cleared by 60 min. Particularly, 84% of **23** was cleared and represents one of the best 2:60 min ratios at that point in time. Despite this promising pharmacokinetic property, this class of compounds was never revisited.

**Table 2:** *In vitro* and *ex vivo* analysis of fused (hetero)cyclic radiotracer candidates in Figure 5.

						**% Injected dose/gram (%ID/g)**		
**Compound**		***n***	**R**	**K_i_ (nM)**	**Log P**	**2 min**	**60 min**	**Ratio^a^**
**22**				n.d.	2.77 ± 0.04	0.64 ± 0.07	0.23 ± 0.23	2.78
**23**				n.d.	2.23 ± 0.04	0.79 ± 0.12	0.11 ± 0.04	7.18
**24**				n.d.	n.d.		n.d.	
**25**				n.d.	n.d.		n.d.	
**26**				3.65 ± 0.49 μM	1.70 ± 0.02^b^	0.65 ± 0.09	0.36 ± 0.06	1.81
**27**				4.64 ± 0.77 μM	1.89 ± 0.04^b^	0.28 ± 0.03	0.12 ± 0.02	2.33
**28**	**a**		NH_2_	n.d.	n.d.		n.d.	
	**b**		NHCH_3_	296	n.d.		n.d.	
	**c**		NH(CH_3_)_2_	53.7	2.45	0.88	0.25	3.52
**29**	**a**	2		33.2^c^	1.08 ± 0.02	0.78 ± 0.07	0.09 ± 0.03	8.67
	**b**	3		102.5^c^	1.10 ± 0.06	0.86 ± 0.07	0.12 ± 0.02	7.17

n.d., not determined.

a Clearance ratio of 60 min/2 min.

b Log D.

c IC_50_.

Using a unique, neutral [^99m^Tc(CO)_2_NO] core, Yang et al. labeled the flavonol moiety with iminodiacetate chelators to give **24** and **25** (74). This technetium core has a formal 2+ charge and forms neutral complexes with iminodiacetate. These two compounds were not evaluated beyond autoradiography as results using transgenic mouse tissue (C57 APP/PS1, 12 months, no sex given) found no binding to amyloid.

In another report from Beijing Normal University, two naphthylethylidene derivatives selected for radiolabeling with ^99m^Tc due to similarities to PET radiotracers [^18^F]FDDNP and [^18^F]FENE (75). Choosing this core is surprising as these radiotracers are known to have affinity for both Aβ and neurofibrillary tangles. Moreover, the ^18^F-labeled compounds have low affinity for Aβ aggregates with K_i_ values in the 400–470 nM range. The group also chose to use a MAMA chelator despite precedent of higher non-specific binding and lower brain uptake. After coordinating Re, the binding affinity of **26** and **27** were found to be 10-fold lower (3–5 μM range) but retained some selectivity for Aβ in brain tissue slices of transgenic mice (C57BL6 APP_swe_/PSEN1, 12 months, no sex given). Low molecular weight and lipophilicities in the ideal range held promise for brain uptake but virtually none was observed in *ex vivo* biodistribution (ICR mice, no age or sex given). High non-specific binding, as indicated by poor clearance at 60 min p.i., was also seen. Unsurprisingly, no follow up studies were conducted using the DDNP or ENE backbones.

Building upon early success with [^123^I]ABC577, the Kyoto group reported three phenylquinazoline derivatives conjugated with BAT-^99m^Tc chelates (76). This scaffold was report to have higher binding affinity and more selectivity for Aβ than other commonly employed PET probes (i.e., PiB, florbetapir). Adding the three carbon linker and Re chelate did lower the binding affinity significantly. Amyloid aggregate binding remained for the dimethyl derivative **28c** but no binding was observed for the other two. Although autoradiographic experiments showed promising co-staining with **28c** and thioflavin-S, low initial brain uptake and moderate washout in healthy male DDY mice (no age given). The authors hypothesized the high stomach uptake pointed to decomposition of **28c** as contributing to low brain uptake.

Two novel 2-aryl imidazole[2,1-b]benzothiazole (IBT; **29**) derivatives were synthesized and successfully labeled with ^99m^Tc radionuclide at high radiochemical purity using fac-[^99m^Tc(CO)_3_(H_2_O)_3_]^+^ synthon (77). Autoradiography and staining with Congo Red confirmed that these complexes bind to Aβ plaques in brain sections of diseased rat models (male Wistar, stereotactic injection with Aβ oligomers at 3 months), but the age of the rats at the time of tissue collection is not given. Both candidates exhibited moderate affinity toward β-amyloid aggregates with IC_50_ values of 33.2 and 102.5 nM in Aβ aggregates. Unlike most of the studies presented in this review, blocking studies were conducted with Aβ_1−42_ aggregate binding to demonstrate some selectivity. In the inhibition assay results, **27b** was found to have higher binding and to be more selectivity. It should be noted that the higher IC_50_ values represent displacement of a fixed amount of ^99m^Tc-labeled compounds by BTA-1 (benzothiazole derivative akin to thioflavin-S) and therefore cannot be directly compared to the K_i_ values obtained from previous studies. As the length of the linker (alkyl chain) increased, so did the IC_50_ value, the lipophilicity, and the initial brain uptake. Although both **29ab** stable in saline after 2 h (92% of both remained intact), no histidine or cysteine challenges were completed. Nevertheless, both **29a** and **b** showed little initial brain uptake and low non-specific binding in biodistribution studies (BALB-C, no sex or age given) which was indicated by the clearance ratios of 8.67 and 7.17, respectively. The relatively high hydrophilicity and molecular weight (>600 Da) may have led to the blunted brain uptake.

### Stilbenes, chalcones, and curcumin derivatives

**Figure 6:**
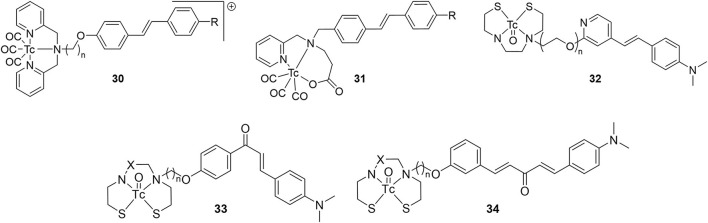
Chemical scaffolds of ^99m^Tc-labeled stilbene, chalcone, and curcumin-like derivatives derivatives for Aβ plaque imaging.

Following the FDA approval of florbetapir in 2012, investigators embarked on developing ^99m^Tc-labeled stilbene analogs (Figure 6; Table 3). The first attempt from Jia et al. gave **30a** and **30b** as the second half of a 2014 study, which included **16** above (64). Only the latter had a strong binding affinity to Aβ_1−42_ aggregates and was radiolabeled. As the aim of this study was to develop a BBB impermeable radiotracer for CAA, it was favorable that **30b** was found to have low initial brain uptake with minor non-specific binding after 60 min. With a reasonably high binding affinity and desired low brain uptake, it is somewhat surprising no follow up studies have been reported.

**Table 3:** *In vitro* and *ex vivo* evaluation of stilbenes 30–32.

						**% Injected dose/gram (%ID/g)**		
**Compound**		***n***	**R**	**K_i_ (nM)**	**Log P**	**2 min**	**60 min**	**Ratio^a^**
**30**	**a**	3	N(CH_3_)_2_	366 ± 70	n.d.		n.d.	
	**b**	5	N(CH_3_)_2_	78 ± 13	1.79 ± 0.14	0.24 ± 0.02	0.10 ± 0.04	2.40
	**c**	1	H	n.d.	1.8		n.d.	
	**d**	1	N(CH_3_)_2_	n.d.	1.0		n.d.	
**31**	**a**	1	H	n.d.	2.1		n.d.	
	**b**	1	N(CH_3_)_2_	n.d.	1.7	0.25 ± 0.05	0.21 ± 0.02	1.19^b^
						0.24 ± 0.02	0.19 ± 0.01	1.26^b^^,^^c^
**32**	**a**	1		13.4 ± 3.3	3.04 ± 0.02	2.10 ± 0.22	0.61 ± 0.08	3.44
	**b**	2		142.1 ± 24.4	3.15 ± 0.01	1.10 ± 0.11	0.15 ± 0.02	7.33
**33**	**a**	5	CO	n.d.	2.55 ± 0.19	0.22 ± 0.05	0.11 ± 0.01	2.00
	**b**	5	CH2	n.d.	2.73 ± 0.16	0.78 ± 0.16	0.16 ± 0.01	4.88
	**c**	3	CO	n.d.	1.51 ± 0.09	0.62 ± 0.27	0.16 ± 0.08	3.88
	**d**	3	CH3	n.d.	2.51 ± 0.05	1.48 ± 0.44	0.17 ± 0.06	8.71
**34**	**a**	3	CH_2_	24.7 ± 6.1	3.36 ± 0.08	0.49 ± 0.08	0.08 ± 0.01	6.13
	**b**	5	CH_2_	13.6 ± 7.8	3.17 ± 0.10	0.47 ± 0.11	0.12 ± 0.02	3.92
	**c**	3	CO	120.9 ± 4.3	3.52 ± 0.10	0.48 ± 0.06	0.09 ± 0.01	5.33
	**d**	5	CO	59.1 ± 24.0	3.57 ± 0.15	0.31 ± 0.06	0.15 ± 0.02	2.07

n.d., not determined.

a Clearance ratio of 60 min/2 min.

b 30/2 min.

c APP/PS1 transgenic mice.

A year later, a team from Australia prepared analogous complexes **30c, 30d**, and **31** (78). A chemistry focused paper, there was little *in vitro* evaluation of these four candidates but there was a rare example of *ex vivo* biodistribution data from a transgenic mouse model (APP/PS1, 10 months, no sex given). There was little brain uptake of **31b** with no statistical difference between wild type and transgenic mice. The exchange of a pyridyl for an acetate group eliminated the positive charge of compounds **30** but low BBB permeability persisted. This paper also reported the first example of small animal SPECT imaging of ^99m^Tc-based AD radiotracers. With **30d** and transgenic mice (APP/PS1, 15 months, no sex given), the activity was found to mostly accumulate in the bladder, liver, and kidney. No brain uptake was observed and there was some accumulation in the thyroid, pointing to possible oxidative degradation to pertechnetate.

In the same studying reporting **12**, Zhang et al. introduced an oligoethyleneoxy linker between ^99m^Tc/Re-BAT and a β-amyloid binding styrene scaffold (61). They intended to improve brain uptake by decreasing the hydrophobicity and replacing the alkyl linker between the targeting vector and chelator with PEG chains. Compound **32a** had the highest binding affinity to Aβ plaques (K_i_ = 13.4 nM). Furthermore, the capability of both **32** analogs to bind Aβ plaques was positively verified by *in vitro* fluorescent staining with transgenic mice (C57BL6 APP_swe_/PSEN1, 12 months, no sex given) and AD patient tissue samples. Similarly, *ex vivo* autoradiography showcased amyloid binding in transgenic mice (but not wild type), matching thioflavin S staining on adjacent slices. Compounds **32a** had high binding affinity and low clearance ratio, while compounds **32b** had lower binding affinity but higher clearance ratios (for reference, [^125^I]IMPY had a K_i_ of 12.5 ± 1.4 nM). Extending the linker length therefore had a mixed result, further illustrating the difficulty in attaining a delicate balance between the pharmacokinetic parameters. Furthermore, the biodistribution in healthy mice (ICR, male, no age given) showed less uptake than compounds **12** on average, but the clearance ratios were better, possibly indicating less non-specific binding. Compound **32b** had a higher uptake than the previous study (with a clearance ratio of 7.33), indicating some potential as a SPECT probe for imaging AD. As with **11d**, **32a** was used *in vivo* with SPECT imaging of a non-human primate (rhesus monkeys, *n* = 6, 3/3 male/female, 4–30 years old). Brains were scanned for 60 min to show moderate brain uptake with little washout. The activity peaked at 20 min with 2–2.6 %ID/g (semi-quantitative) with clearance ratios around 1.4. This uptake is higher than for **11d** but much lower than the PET analogs. That said, there were no Aβ plaques present in these animals and further investigation into these promising candidates may be warranted.

Published in 2010 following their first flavone study, Ono et al. reported four chalcone derivatives with **33** as a scaffold (79). These were conjugated to BAT and MAMA through either a three or five methylene linker (Figure 6; Table 3). Of the four, **33d** had the lowest binding to Aβ_1−42_ aggregates but had by far the best pharmacokinetics (highest initial uptake, best clearance). Using the same mice as their previous study (Tg2567, 30-months-old, female), brain sections were stained with similar intensity for all four derivatives (matched thioflavin-S staining pattern). Given the *ex vivo* and *in vitro* results, there appears to be a disconnect between solution Aβ_1−42_ aggregate binding affinity and the ideal pharmacokinetics for imaging. Perhaps this was why the authors did not determine the K_i_ values for these compounds or for **23** and **24**.

Four ^99m^Tc-labeled dibenzylideneacetone derivatives (80) were successfully synthesized and biologically evaluated. This backbone was selected due to its similarity to curcumin (81). Previous work had shown that the para position of the phenoxy ring had a high tolerance for bulky substitutions. Both BAT and MAMA chelators were conjugated and radiolabeled. Complexes **34** showed high to moderate affinity for Aβ_1−42_ aggregates (K_i_ = 13.6 – 120.9 nM; [^125^I]IMPY = 11.5 ± 2.5 nM), and selectively stained β-amyloid plaques on brain sections of transgenic mice (C57BL6 APP_swe_/PSEN1, 11 months, no sex given). *In vitro* fluorescent staining confirmed the presence and distribution of β-amyloid plaques using thioflavin-S as well as absence of staining in age-matched control mice tissue. Biodistribution in normal mice (ICR, 5 weeks, male) revealed that **34b** and **d** exhibit moderate clearance ratios, while **34a** and **34c** exhibit higher clearance ratios. Once again, the MAMA based ligand showed a higher non-specific binding profile compared to BAT. Unfortunately, the initial uptake was low for all compounds tested. Additional refinements are needed to improve the penetration through BBB, but these promising results indicate that **34a** may be a potential SPECT probe. It has a high affinity for β-amyloid plaques and an excellent clearance ratio; therefore, it is the most suitable derivative from this series for further attention.

### Bivalent Tc-99m ligands for AD

**Figure 7:**
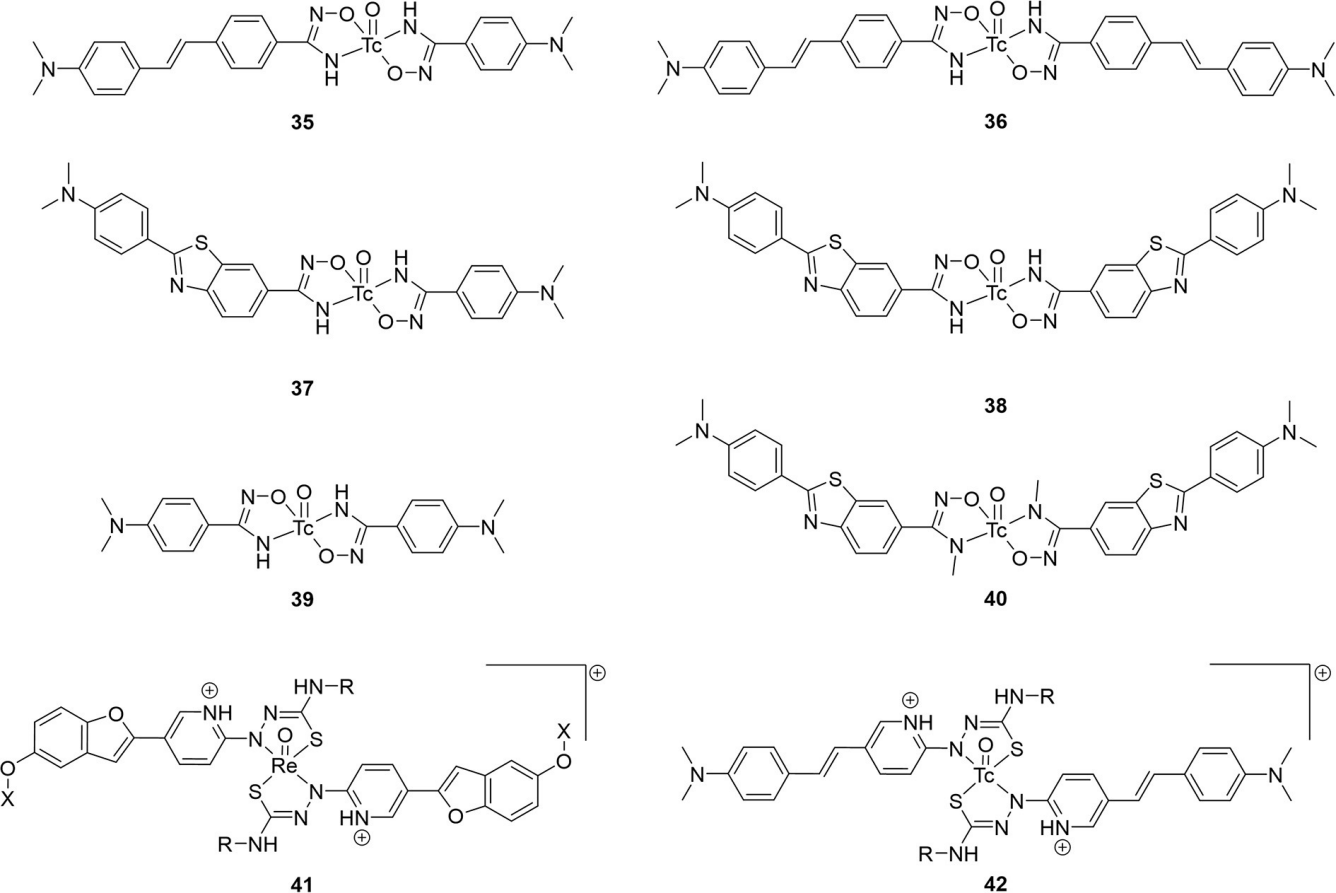
Chemical scaffolds of ^99m^Tc-labeled of bivalent Aβ binding SPECT radiotracers.

High binding affinity is seen as a prerequisite for developing useful molecular imaging probes, although some results within this review may challenge this assumption, at least for imaging Aβ aggregates. Nonetheless, multivalent ligands are known to have enhanced binding compared with monovalent counterparts (82). The enhanced binding of bivalent ligands may arise from rebinding of the ligand prior to complete dissociation. Another explanation is multisite binding, however the distance between targeting vectors may be too short in the cases reviewed herein. The Kyoto group built upon their previous work with ^99m^Tc-hydroxamamide complexes, shown to have high kinetic stability, to prepare 1:2 metal to ligand complexes **35**–**39** (Figure 7; Table 4) (83). These were easily radiolabeled in high yield and two isomers were isolated as previously reported with ^99m^Tc-hydroxamamides. The absolute configurations were not determined but the isomers with shorter retention times on the HPLC were designated A and the latter as B. In the solution based Aβ_1−42_ aggregate binding assay, only the A isomers were used. These assays showed the bivalent ligands had stronger binding with **36** and **38** having greater affinity compared to **35** and **37**, respectively. In the case where no amyloid targeting vector was present (**39**), no binding was observed.

**Table 4:** *In vitro* and *ex vivo* data for bivalent Aβ binding SPECT radiotracers.

					**% Injected dose/gram (%ID/g)**		
**Compound** ^**a**^		**R**	**X**	**IC_50_ (μM)**	**2 min**	**60 min**	**Ratio^b^**
**35**	**A**			0.72 ± 0.10	0.37 ± 0.05	0.08 ± 0.00	4.62
	**B**			0.38 ± 0.08		n.d.	
**36**	**A**			16.40 ± 2.47	0.28 ± 0.03	0.11 ± 0.01	2.55
	**B**			2.55 ± 0.45		n.d.	
**37**	**A**			0.26 ± 0.02		n.d.	
	**B**			0.47 ± 0.05	0.36 ± 0.03	0.08 ± 0.02	4.50
**38**	**A**			2.80 ± 0.32		n.d.	
	**B**			5.78 ± 0.53	0.37 ± 0.11	0.05 ±0.01	7.40
**39**				n.d.		n.d.	
**40**				0.56 ± 0.08	0.35 ± 0.04	0.11 ± 0.01	3.18
**41**	**a**	CH_3_	CH_3_	n.d.		n.d.	
	**b**	C_2_H_4_N(CH_3_)_2_	CH_3_	n.d.		n.d.	
	**c**	CH_3_	H	n.d.		n.d.	
	**d**	C_2_H_4_N(CH_3_)_2_	H	n.d.		n.d.	
**42**	**a**	CH_3_		n.d.		n.d.	
	**b**	C_2_H_4_N(CH_3_)_2_		n.d.		n.d.	

n.d., not determined.

a Capital letter apply to undetermined isomers of **35**–**39** with “A” eluting first on HPLC.

b Clearance ratio of 60/2 min.

They next evaluated the inhibitory binding by displacing **35**–**39** from Aβ_1−42_ aggregates with unlabeled PiB, thus higher values indicate stronger binding. Trends from the assay results matched their previous assay, with no binding of **39** seen. Interestingly, the A isomer of **35** and **36** showed stronger binding whereas the opposite was observed for **37** and **38**. Finally, *in vitro* autoradiography comparing radiotracer binding in wild-type (no strain given, female, 28 months) and transgenic mice (Tg2576, female, 28 months) showed (a) higher uptake for all eight compounds in the latter group, (b) good correlation to thioflavin-S staining, and (c) appreciable blocking with unlabeled PiB in transgenic animals. Preliminary biodistribution studies with the highest affinity compound, **36**, showed very low initial uptake (0.28 %ID/g).

Given the low brain uptake with the bivalent compounds, the same group published two more studies investigating their utility for imaging CAA (84, 85). As Aβ_1−40_ is the predominate subtype of amyloid in this disease, Iikuni et al. measured the affinity of **35**–**38** to these aggregates (84). After assessing brain permeability compared to a prototypical PET agent, [^18^F]florbetapir, low uptake led to the conclusion **35**–**38** would be well suited for this type of amyloidosis. More of this approach, directly comparing putative SPECT radiotracers to clinical PET agents, should be conducted with future studies. Another rare experiment from this study was *in vivo* SPECT/CT with Tg2576 mice and wild-type mice (female, 27 or 30 months) 30 min p.i. Although specific binding was observed in autoradiography studies with brain tissue from these rodents, there was no differential distribution observed *in vivo* between transgenic and wild-type mice with **36A**. This could be due to high blood:brain ratio observed in the *ex vivo* studies (50:1–60:1). The contribution from the non-specific binding in plasma could be overwhelming the small signal from cerebral amyloid. Further optimization is required for *in vivo* imaging cerebral amyloid angiopathy.

To avoid obtaining two geometric isomers upon chelation of ^99m^Tc, the authors used a methylated hydroxamamide (85). Previous studies also noted this alteration increased stability (86). Herein, methylating **38** led to **40** with a marked increase of stability in mouse plasma (ddY, male, 5 weeks) from 86 to 93% radiochemical purity at 2 h. Removing the hydrogen bonding amine group did not improve brain permeability. Both **38** and **40** had circa 0.35 %ID/g in ddY mice at 2 min p.i. Binding affinity was negatively impacted by the methyl groups with the IC_50_ values of PiB displacement dropping to 0.56 μM. *In vitro* autoradiography revealed significantly higher non-specific binding to Tg2576 (female, 28 months) brain sections. This contrasted with distinctive labeling of Aβ plaques with **38**. Similar outcomes were observed with human CAA patient brain tissues.

In a report focused on chemistry and radiolabeling, Fletcher et al. provided details for six bivalent thiosemicarbazide ligands **41** and **42** (87). The sole biological evaluation was the interaction of the ligands with Aβ_1−42_ in human tissue samples. The fluorescent signal from Thioflavin-T would decrease with respect to increased ligand binding. It was found that the **41d** had the greatest signal reduction, followed by **42b**, **41c**, **41b**, **42a**, and the lowest reduction was **41a**. It appears the phenolic and amino groups present on the benzofuran and stilbene groups had a positive benefit to binding affinity. Only the stilbenes (**42**) were radiolabeled with ^99m^Tc but no further biological evaluation was conducted.

## Integrated Tc-99m Aβ probes

Radiometalated PET or SPECT probes fall into three categories: (1) colloids, (2) conjugated, such as those discuss above, and (3) metal-centric or integrated small molecules. By making the metal an integral part of the scaffold and target binding motif, the molecular weight, volume, and lipophilicity (from linkers and chelators) can be minimized. The examples below include the most promising ^99m^Tc-based amyloid radiotracers reported with respect to initial brain uptake and clearance after 60 or 90 min p.i. They often possess lower *in vitro* binding affinity, but this may not be such as important factor for *in vivo* interactions with Aβ-sheets.

### Benzo–thiazole and –imidazole

**Figure 8:**
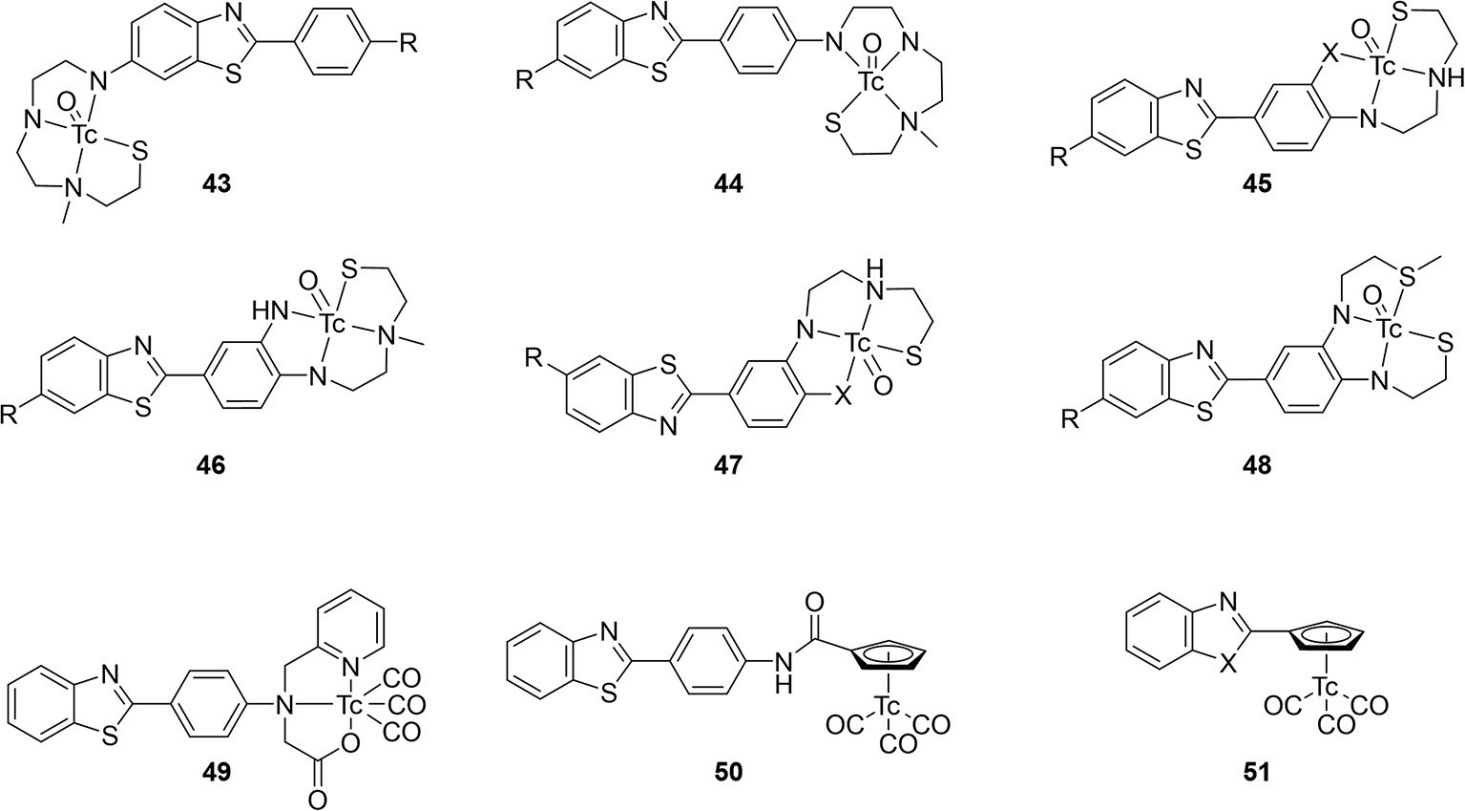
Integrated chemical scaffolds of ^99m^Tc-labeled benzothiazole derivatives for imaging Aβ plaques.

Starting from 2009, a study from the inventors of [^11^C]PiB (first PET amyloid radiotracer to be widely used), several ^99m^Tc-based candidates, **43**–**48**, were reported (Figure 8; Table 5) (57, 88). The integrated approach was adopted for these as a conjugated analog, **8**, was thought to be too lipophilic (Log P = 3.26) and heavy (MW = 617) for good BBB permeability. Integrating the tetradentate chelators into the pharmacophore would address these concerns. Additionally, by placing these groups at the 6 and 4′ positions, their electron donating effects will promote higher binding affinity. The weights were decreased to <550 Da and the lipophilicities now ranged from 1.21 to 2.59; within the ideal range. In the initial study (**43a**, **44ab**, and **45ab**) the K_i_ values were reasonable for imaging amyloid (29.7–88.6 nM), albeit lower than the **8** (10 nM). The follow-up study in 2013 was by far the most extensive structure activity relationship investigation in this area of research. In total, an additional 21 compounds were prepared and evaluated for lipophilicity and binding affinity (Table 5). By varying the heteroatoms of the chelator, the lipophilicity was finetuned. For example, switching from an SN_2_O chelator to SN_2_S raised the lipophilicity of the Re chelates an average of 0.75 (e.g., **45a** vs. **45b**). With respect to binding affinity, it was generally found that including a fluorine or methoxy donating group enhanced binding affinity. The position of the chelators also had a significant impact on affinity. The study authors found a 2–7-fold increase when a semi-rigid chelator was attached in the 4′ vs. 3′ position (**45** vs. **47**). Although one of the largest efforts in this area, no Tc labeling was completed along with no *ex vivo* biodistribution experiments to confirm brain uptake.

**Table 5:** *In vitro* and *ex vivo* data for integrated ^99m^Tc probes in Figure 8.

					**% Injected dose/gram (%ID/g)**		
**Compound**		**X/R**	**K_i_ (nM)**	**Log P**	**2 min**	**60 min**	**Ratio^a^**
**43**	**a**	N(CH_3_)_2_	30	2.59		n.d.	
	**b**	H	556	2.54		n.d.	
	**c**	F	617	2.67		n.d.	
	**d**	OCH_3_	85	2.37		n.d.	
**44**	**a**	OH	89	1.21		n.d.	
	**b**	${\text{OCH}}_{3}^{\text{b}}$	87	2.52		n.d.	
	**c**	H	378	2.65		n.d.	
	**d**	F	118	2.84		n.d.	
**45**	**a**	O/OCH_3_	30	1.65		n.d.	
	**b**	S/OCH_3_	43	2.30		n.d.	
	**c**	O/H	109	1.70		n.d.	
	**d**	O/F	64	1.90		n.d.	
	**e**	O/OCH_3_	30	1.65		n.d.	
	**f**	S/H	38	2.41		n.d.	
	**g**	S/F	31	2.60		n.d.	
	**h**	S/OCH_3_	43	2.30		n.d.	
**46**	**a**	N/H	90	2.333		n.d.	
	**b**	N/F	113	2.50		n.d.	
	**c**	N/OCH_3_	61	2.21		n.d.	
**47**	**a**	O/H	280	1.68		n.d.	
	**b**	O/F	226	1.87		n.d.	
	**c**	O/OCH_3_	140	1.59		n.d.	
	**d**	S/H	264	2.49		n.d.	
	**e**	S/F	93	2.65		n.d.	
	**f**	S/OCH_3_	132	2.45		n.d.	
**48**	**a**	H	200	2.35		n.d.	
	**b**	F	148	3.53		n.d.	
	**c**	OCH_3_	178	3.25		n.d.	
**49**			> 30 mM	1.03 ± 0.01	0.25 ± 0.04	0.11 ± 0.01	2.27
**50**			13.6 ± 4.8	2.35 ± 0.08	0.53 ± 0.11	0.25 ± 0.08^c^	2.12^c^
					0.52 ±0.08^d^	1.94 ± 0.25^c^^,^^d^	
**51**	**a**	S	65.8 ± 21.3	2.52 ± 0.14	7.94 ± 1.46	0.20 ± 0.03^c^	39.7^c^
	**b**	NH	7.0 ± 2.9	1.84 ±0.17	3.99 ± 0.60	0.04 ± 0.01^c^	99.8^c^
	**c**	NCH_3_	5.7 ± 2.9	1.50 ± 0.12	5.36 ± 0.65	0.09 ± 0.02^c^	59.6^c^

n.d., not determined.

a Clearance ratio of 60/2 min.

b Repeated in Pan et al. (88).

c 90 min.

d In Tg mice.

Hoping to take advantage of the kinetic inertness of the ^99m^Tc(CO)_3_ core (89), Sapati et al. produced the neutral compound **49**. Radiolabeling was highly efficient (95% radiochemical yield) and was found to be stable in serum for up to 6 h. Histidine and cysteine challenges likewise caused no degradation of **49**. No IC_50_ or K_i_ values were given for this compound. By mixing 0.05 nM of radiotracer with 20 μM of Aβ_1−40_ peptide, 4.6 ± 0.3% of **49** was found to specifically bind. To assess specificity, thioflavin T was used to inhibition roughly 25% of that initial binding at 10 and 30 mM. With the lipophilicity near the bottom of the ideal range, **49** was evaluated in normal mice (no strain, age, or sex given). Little change to uptake in the gastrointestinal tract after 2 h p.i. further demonstrated the stability of **49**. Unfortunately, brain uptake was minimal and clearance after 1 h was poor (Table 5).

As previously mentioned for **9**, the cyclopentadienyl piano-stool chelates are compact, moderately lipophilic groups (90). A preliminary study affixing this chelate to the anilino group showed excellent binding affinity despite the metal center being integrated into the pharmacophore (91). The rhenium congener of **50** was also observed to co-stain areas of human AD brain tissue with thioflavin S using confocal microscopy. After demonstrating good stability in saline and under transchelation challenges (>95 and >90% after 6 h, respectively), brain uptake was evaluated. Biodistributions studies in normal mice (Swiss albino, 5 weeks, no sex given) showed low initial uptake and poor clearance after 90 min. In a rare *ex vivo* biodistribution experiment in transgenic animals (C57BL6 5xFAD, 7 months, no sex given), the initial uptake was the same but there was a 4-fold increase in uptake after 90 min. Static gamma camera images also clearly showed brain accumulation of **50**. Increase uptake was attributed to the presence of confirmed amyloid plaques (co-staining post-mortem tissue). This study demonstrated that an effective SPECT radiotracer will depend on an intricate balance of factors and the importance of including disease models in their evaluation.

Building on their promising study, the group from Demokritos Institute prepared another set of candidates wherein the anilino group was replaced the Cp^99m^Tc(CO)_3_ core (so-called cytectrenes) (92). Compounds **51** showed remarkable brain uptake and clearance in normal mice (Swiss albino, 5 weeks, male). These were by far the highest uptake observed at 2 min p.i. and greatest clearance (Table 5). Initial uptake of **51a** was on par with [^18^F]florbetapir (7.33 %ID/g in mice) (70) and was greater than [^18^F]florbetaben (4.77 %ID/g) (93). More remarkable still was the high affinity for **51b** and **51c**, further emphasizing the minimal impact this ^99m^Tc chelate has on biological activity. Further biodistribution studies of **51a** in transgenic mice (C57BL6 5xFAD, 11 months, no sex) showed higher accumulation at 15 min p.i. compared to their wild-type littermates (3.90 ± 0.19 vs. 2.68 ± 0.06, respectively). Staining of brain tissue from the transgenic group with thioflavin S confirmed the presence of amyloid plaques, however, no co-staining or autoradiography with **51a** was reported. Anti-amyloid aggregation activity of the three Re congeners was also evaluated but is outside of the scope of this review.

### Miscellaneous integrated radiotracers

**Figure 9:**
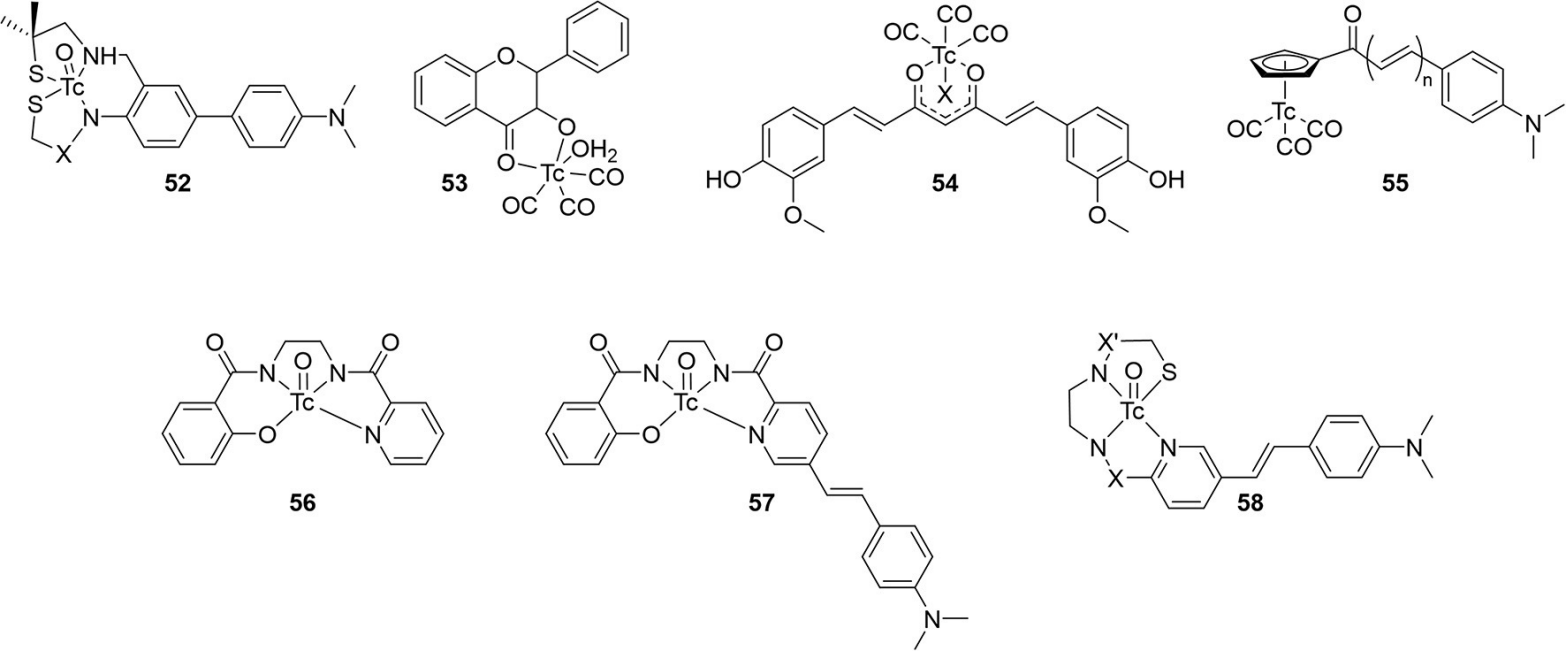
Integrated chemical scaffold of non-benzothiazole based ^99m^Tc-labeled radiotracer for imaging Aβ plaques.

Seven other integrated compounds with various pharmacophores were reported (Figure 9; Table 6). The biphenyl **52** was an early example from the University of Pennsylvania. They incorporated a MAMA– or BAT–like ligand into one anilino ring but these were not able to adequately enter the brain of mice (ICR, 2–3 months, male). The clearance after 60 min was also low, indicating some non-specific binding. Another detriment to **52** was a side reaction upon coordination. An imine formed a much more lipophilic product which, although easily removed, decreased the radiochemical yield.

**Table 6:** *In vitro* and *ex vivo* data for compounds in Figure 9.

					**% Injected dose/gram (%ID/g)**		
**Compound**		**X/R**	**K_i_ (nM)**	**Log P**	**2 min**	**60 min**	**Ratio^a^**
**52**	**a**	CO	n.d.	2.23	0.29 ± 0.04	0.19 ± 0.03	1.53
	**b**	CH_2_	n.d.	2.57	1.18 ± 0.20	0.46 ± 0.08	2.57
**53**			11.16^b^	n.d.	0.48 ± 0.05	0.44 ± 0.08	1.09
**54**	**a**	H_2_O	n.d.	n.d.		n.d.	
	**b**	imidazole	n.d.	n.d.		n.d.	
	**c**	Isocyanocyclo-hexane	n.d.	n.d.		n.d.	
**55**	**a**	*n* = 1	899 ± 78	2.89 ± 0.09	4.10 ± 0.38	0.50 ± 0.08	8.20
	**b**	*n* = 2	211 ± 19	3.61 ± 0.04	2.30 ± 0.27	0.55 ± 0.08	4.18
	**c**	*n* = 3	108 ± 16	3.45 ± 0.09	1.11 ± 0.34	0.51 ± 0.08	2.18
**56**			n.d.	1.44 ± 0.02		n.d.	
**57**			855	1.87 ± 0.02		n.d.	
**58**	**a**	X=X′=CH_2_	260 ± 82	1.54 ± 0.03	0.15 ± 0.06	0.17 ± 0.01	0.88
	**b**	X=CH_2_; X′=CO	241 ± 46	2.04 ± 0.07	0.36 ± 0.09	0.15 ± 0.02	2.4
	**c**	X=X′=CO	260 ± 73	n.d.		n.d.	

n.d., not determined.

a Clearance ratio of 60 min/2 min.

b K_d_.

Along with conjugated examples **24** and **25**, Yang et al. prepared **53**. This flavone-like complex had similar binding affinity to reported fluorescent dyes and, unlike the former two candidates, **53** was able to stain Aβ aggregates in tissue samples from transgenic mice (C57 APP-PS1, 12 months, no sex given). In biodistribution studies, the low initial uptake was not cleared from the brain (Table 6). Both the poor uptake and high non-specific binding could be due to the labile water ligand ^99m^Tc. If replaced in the blood pool, **53** may become charged and not enter the brain. Likewise, binding to nucleophilic peptide residues could lead to high retention to parenchyma.

Combining the stable d^6^ core of ^99m^Tc(CO)_3_ with a curcumin pharmacophore led to candidate radiotracer **54**. Sagnou et al. labeled the β-diketone with various monodentate co-ligands. The initial water complex **54a** was readily prepared (90%) with moderate and excellent ligand exchange for imidazole (25–35%) and isocyanocyclohexane (90%), respectively. As a chemistry focused paper, little biological evaluation was completed. Staining of adjacent slices from human post-mortem tissue showed co-localization of all three derivatives with curcumin. No further studies with this compound class were reported.

Returning to the piano stool Cp^99m^Tc(CO)_3_ core, the group at Beijing Normal University prepared chalcone derivatives **55** with 1–3 unsaturated groups. Although still an extended pi system, the flexibility of these groups vs. aryl rings may have contributed to the poor binding affinity ranging from 100 to 900 nM. Each of the three **55** derivatives selectively stained Aβ aggregates in tissue from transgenic mice (C57BL6 APPswe/PSEN1, 11 months, male) and a human AD patient. Moreover, measured lipophilicity values were within or near the ideal range for brain permeation. Biodistribution in normal mice (ICR, 5 weeks, male) showed an inverse relationship between brain uptake and molecular weight or lipophilicity. The uptake and clearance are remarkably higher than bifunctional conjugate analogs like **33**. Unfortunately, blocking of PgP with cyclosporin A increased the brain uptake (1.5–1.6-fold), indicating **55** may be a substrate for this efflux transporter.

Compounds **56** and **57** were reported by Hayne et al. to evaluate neutral [TcO]^3+^ core integrated into a styrylpyridine pharmacophore. The Re analogs were crystallized and x-ray crystallographic analysis showed a distorted square-pyramidal environment around the metal center. As planar structures are desired for high Aβ aggregate binding, this orientation may harm affinity. Nonetheless, both compounds were radiolabeled with ^99m^Tc in >90 and 60% radiochemical yield for **56** and **57**, respectively. The stability of **57** was not reported and **56** was shown to be stable to glutathione exchange (<5% decomposition after 1 h) but not histidine (16% hydrophilic species after 1 h). As a chemistry focused paper, the biological evaluation was limited to Aβ aggregate binding and confocal microscopy of **57**. As with other compounds, co-staining of adjacent slices of post-mortem tissue samples from confirmed AD patients was observed. More biological evaluation may not be justified given the low stability to the histidine challenge, poor radiochemical yield, and low binding affinity.

In the most recent report covered in this review, Spyrou et al. outlined the preparation of three derivatives of **58**. These incorporated the stilbene backbone with an N_3_S chelator for oxotechnetium (V) but replaced the phenolate group of **57** with a thiolate to improve the stability and lower lipophilicity. Both amide and amine donors were prepared with improved stability of both (>94% parent of both after 30 min). All three Re complexes of **58** had similar binding affinity for Aβ_1−42_. Compound **58c** could not be isolated in sufficient purity for *ex vivo* experiments, however, neither **58a** or **58b** has sufficient initial brain uptake or good clearance.

## Future perspective

A diverse array of over fifty ^99m^Tc-labeled scaffolds has been reported, however, with few promising candidates for clinical translation. There is minimal correlation between binding affinity and favorable pharmacokinetics from the reviewed scaffolds. For example, looking at **51** and **55**, there is in fact an inverse relationship between affinity and uptake/clearance properties. Although *in vitro* binding affinity experiments are easy to complete, their utility for selecting candidates for radiolabeling and *in vivo*/*ex vivo* experiments may be overvalued. Furthermore, co-staining experiments with validated histochemical amyloid dyes and novel radiotracer candidates (Re-congener fluorescence or autoradiography) rarely disagreed. While this *in vitro* data remains valuable, to significantly advance ^99m^Tc-based radiotracers for brain SPECT imaging, “failing faster” in whole organisms (e.g., animals) may be warranted. The addition of predictive *in silico* methods for predicting brain permeability may offer the most cost effective, early screen for BBB permeability (94, 95). Better understanding of the structure-activity relationship for both BBB permeability and target affinity is required to build those models and should be a priority over cherry-picking. Trends from the compounds discussed herein point to the integrated approach finding more success.

The focus also needs to shift away from *ex vivo* studies evaluating initial uptake and clearance in healthy animals. Whole body imaging in AD disease model animals and age-matched controls would better and more rapidly screen potential candidates. Adding dynamic imaging with proper plasma metabolite analysis, like PET radiotracer development, will provide more details into the pharmacokinetics and assist with future iterations of SPECT radiotracer development. New SPECT systems designed for animal imaging have improved spatial resolution and sensitivity such that longitudinal studies in transgenic rodents, particularly rat models (96), should be possible to track sensitivity of ^99m^Tc-based radiotracers to changes in amyloid plaques. Clinical translation could be further accelerated by shifting away from rodents into porcine models (less costly and fewer ethic concerns than non-human primates) (97). Their larger brains can be imaged using clinical SPECT scanners (easy translation to humans) and will permit more granular assessment of radiotracer distribution. Continued confirmation of target specificity through histochemistry and autoradiography in animal and human tissues samples will affirm *in vivo* results. While there was improvement over time to reporting species and sex of animals, studies with both sexes need to become standard practice (98).

A final concern translating SPECT probes into the clinic could be white matter binding. Not only does this “non-specific” binding decrease signal to noise, the partial volume effect from white matter will confound quantitative analysis. Fluorine-18 radiotracer displayed higher subcortical white matter uptake compared to [^11^C]PiB, potentially due to increased lipophilicity (99). This reiterates the need for optimizing physiochemical properties during radiotracer development beyond affinity. For amyloid imaging, this uptake may be unavoidable as the so-called non-specific binding has been tied to myelin coating on axons (100). It does point to a potential additional application of ^99m^Tc-based radiotracers for quantifying white matter lesions. With no clear lead candidate radiotracer approaching clinical translation, it could be premature to worry about optimizing signal to noise ratios, but it bears keeping in mind when designing and developing putative ^99m^Tc-based radiotracers.

For SPECT neuroimaging, the ^99m^Tc-based radiotracer development field needs to continue growth to keep pace with other SPECT technological advances. Even in a review extoling the benefits of SPECT/CT (20), there is hardly a mention of neurological applications. Separately, a perspective article on the direction of molecular imaging has no mention of SPECT (21). By changing the perspective of SPECT as an inferior neuroimaging modality, disparate access to leading-edge molecular imaging can be overcome. Indeed, the more widespread and lower cost of SPECT/CT is a wasted resource if only used from the neck down. To move out of the shadow cast by PET for imaging the brain, effective gamma emitting radiotracers are critically needed. By applying some lessons outlined in this review, particularly with remarkable brain penetration of integrated probes, to other targets would expand the radiotracer library for neuro-SPECT (i.e., tau, demyelination, monoamine receptors, etc.). Thus, barriers to accessing nuclear imaging modalities in rural or underdeveloped areas can be removed. Improved access to diagnostic imaging then generates improved health outcomes, further lowering the societal cost burden. This all starts with radiotracer development.

## Author contributions

JH conceived of the project and supervised GT and AK. AK conducted the initial literature review and drafting as part of her senior undergraduate project. GT proofread, completed the thorough literature review, and wrote the manuscript. All authors contributed to the article and approved the submitted version.

## Funding

This work was provided by Western University Internal Research Funds for AK Fourth Year Project, New Frontier's in Research Fund—Exploration (NFRF-E 2020-01021) funds GT training.

## Conflict of interest

The authors declare that the research was conducted in the absence of any commercial or financial relationships that could be construed as a potential conflict of interest.

## Publisher's note

All claims expressed in this article are solely those of the authors and do not necessarily represent those of their affiliated organizations, or those of the publisher, the editors and the reviewers. Any product that may be evaluated in this article, or claim that may be made by its manufacturer, is not guaranteed or endorsed by the publisher.
